# Genetic characterization of the non-structural protein-3 gene of bluetongue virus serotype-2 isolate from India

**DOI:** 10.14202/vetworld.2017.348-352

**Published:** 2017-03-23

**Authors:** Raghavendra Sumanth Pudupakam, Shobana Raghunath, Meghanath Pudupakam, Sreenivasulu Daggupati

**Affiliations:** 1 Department of Veterinary Microbiology, College of Veterinary Science, Sri Venkateswara Veterinary University, Tirupati, Andhra Pradesh, India; 2 Department of Biomedical Sciences and Pathobiology, Virginia Polytechnic Institute and State University, Blacksburg, Virginia, USA; 3 Department of Biochemistry, Osmania University, Hyderabad, Telangana, India

**Keywords:** bluetongue virus, non-structural protein-3 gene, phylogenetic analysis

## Abstract

**Aim:**

Sequence analysis and phylogenetic studies based on non-structural protein-3 (NS3) gene are important in understanding the evolution and epidemiology of bluetongue virus (BTV). This study was aimed at characterizing the NS3 gene sequence of Indian BTV serotype-2 (BTV2) to elucidate its genetic relationship to global BTV isolates.

**Materials and Methods:**

The NS3 gene of BTV2 was amplified from infected BHK-21 cell cultures, cloned and subjected to sequence analysis. The generated NS3 gene sequence was compared with the corresponding sequences of different BTV serotypes across the world, and a phylogenetic relationship was established.

**Results:**

The NS3 gene of BTV2 showed moderate levels of variability in comparison to different BTV serotypes, with nucleotide sequence identities ranging from 81% to 98%. The region showed high sequence homology of 93-99% at amino acid level with various BTV serotypes. The PPXY/PTAP late domain motifs, glycosylation sites, hydrophobic domains, and the amino acid residues critical for virus-host interactions were conserved in NS3 protein. Phylogenetic analysis revealed that BTV isolates segregate into four topotypes and that the Indian BTV2 in subclade IA is closely related to Asian and Australian origin strains.

**Conclusion:**

Analysis of the NS3 gene indicated that Indian BTV2 isolate is closely related to strains from Asia and Australia, suggesting a common origin of infection. Although the pattern of evolution of BTV2 isolate is different from other global isolates, the deduced amino acid sequence of NS3 protein demonstrated high molecular stability.

## Introduction

Bluetongue is an infectious, non-contagious, arthropod-borne viral disease of ruminants caused by bluetongue virus (BTV), an archetypal member of the Orbivirus genus in the family *Reoviridae*. The disease causes substantial economic losses to livestock industry worldwide. In addition, there are mandatory restrictions on the movement of ruminants and other animal products from BTV endemic countries to BTV free countries as it is listed under “List A” diseases by Office Internationale des Epizooties [1]. BTV is architecturally complex and its genome is composed of 10 discrete segments of double-stranded RNA of approximately 19,200 base pairs in length [2,3]. Virus particle is approximately 90 nm in diameter comprising a three-layered icosahedral capsid protein [4,5]. Viral genome encodes for seven structural (VP1 through to VP7) and four non-structural (NS1 through to NS4) proteins [6,7].

At present, 27 BTV serotypes have been reported worldwide [8,9]. Of these, 13 BTV serotypes (Serotypes-1, 2, 3, 4, 6, 9, 10, 12, 16, 17, 18, 21 and 23) were isolated in India [9,10]. The occurrence of new serotypes suggests regular incursions of BTV into India. Global climatic changes and activity of transmission vectors resulted in intercontinental spread of BTV. As a result, significant evolutionary diversity was observed among field strains [11]. Reassortment, genetic drift, and shift were identified as the key factors responsible for the evolution of new BTV serotypes [12]. It is essential to investigate the geographical origin of novel BTVs for a better understanding of their incursions into enzootic foci. This can be achieved by comparative analysis of non-structural protein-3 (NS3) gene [13]. Although isolation and serological characterization of BTV serotype-2 (BTV2) Indian isolate has been reported [14], genetic analysis of NS3 gene and its phylogenetic relationship to global isolates has not been reported so far.

To characterize the NS3 gene of Indian BTV2 isolate, nucleotide and amino acid sequences were analyzed in comparison with those of strains representative of different geographical regions worldwide. Phylogenetic relationship of BTV2 to global isolates has been established.

## Materials and Methods

### Ethical approval

The experiments comply with guidelines laid down by the Institutional Ethical Committee.

### Propagation of virus

BTV2 maintained at the Department of Microbiology, College of Veterinary Science, Tirupati at 2^nd^ passage level, was cultivated in BHK-21 cell line using Glassgow’s Modified Eagles Medium containing 5% fetal calf serum.

### Amplification and cloning of BTV NS3 gene

The identification of the virus was confirmed by amplifying a 762 bp region (20 to 781nt) of the conserved region of S10 (NS3) gene of the virus using reverse transcriptase polymerase chain reaction. Trizol method of RNA extraction was used to extract total RNA from infected cells from 25 cm^2^ tissue culture bottle when the cytopathic effect was about 75%. Primers, NS3L-GCGGGATCCATGCTATCCGGGCTGAT and NS3R-GGCAAGCTTCCCCGTTAGACAGCAGT were used to reverse transcribe and amplify NS3 gene. The reverse transcription was carried out by the addition of five units of avian myeloblastosis virus reverse transcriptase enzyme (Bangalore Genei Pvt. Ltd.). The synthesized cDNA was used to amplify the NS3 gene of BTV by polymerase chain reaction with initial denaturation at 94°C for 3 min, followed by 30 cycles of 1 min denaturation at 94°C, 1 min primer annealing at 58°C and 2 min primer extension at 72°C. The final primer extension was kept at 72°C for 10 min.

The purified NS3 gene product was cloned in pTZ57 R/T cloning vector (Fermentas USA). The recombinant plasmid DNA was transformed into calcium chloride treated DH5α (*Escherichia coli*) competent cells and plated on Luria-Bertani agar medium. The recombinant clones were selected initially by blue–white screening. The selected clones were further confirmed by restriction enzyme digestion analysis and sequencing methods. For sequencing, three clones were selected from several recombinant clones. Sequencing was done in both directions using T7 and M13 primers for all the clones to generate a contiguous sequence using automated DNA sequencer (ABI Perkin Elmer). The sequence has been submitted to NCBI (GenBank) with accessions number KX650180.

### Sequence and phylogenetic analysis

The coding region (690 bp) of NS3 gene was subjected to sequence analysis and compared with different serotypes across the globe. The sequence analysis was performed after converting into FASTA format on internet (website: http://www-ncbi-nlm-nih-gov.ezproxy.lib.vt.edu). Multiple alignments of nucleotide and deduced amino acid sequences were done using CLUSTAL W program. Both percent nucleotide identity and amino acid residue substitutions were studied on comparison with those of different BTV serotypes and isolates of BTV2 from various geographical regions.

The phylogenetic tree was inferred using the neighbor-joining method in MEGA7 (20). The following convention was used to identify nucleotide sequences — BT_serotype number_three-letter country code_ last three digits of sequence accession number as listed in Table-1. The S10 gene sequence of epizootic hemorrhagic disease virus serotype 1 was used as the outgroup to root the trees. The percentage of replicate trees in which the associated taxa clustered together in the bootstrap test (500 replicates) are shown next to the branches. The evolutionary distances were computed using the maximum composite likelihood method and are in the units of the number of base substitutions per site. The analysis involved 46 nucleotide sequences.

**Table-1:** BTV isolates subjected to sequence analysis.

Country	Serotype	Accession number	ID
North America			
USA	10	AF044385	BT10USA385
USA	11	AF044704	BT11USA704
USA	13	AF044710	BT13USA710
USA	17	AF044709	BT17USA709
Central America and Caribbean Basin			
Honduras	1	AY426598	BT1HND598
Costa Rica	3	AY426599	BT3CRI599
Dominican Republic	4	AY426602	BT4DOM602
Honduras	6	AY426603	BT6HND603
Dominican Republic	8	AY426604	BT8DOM604
Jamaica	12	AY426595	BT12JAM595
Puerto Rica	17	AY426596	BT17PRI596
Africa and Mediterranean Basin			
South Africa	1	AF512911	BT1ZAF911
South Africa	2	AF512920	BT2ZAF920
Greece	1	AY677628	BT1GRC628
France	2	AF481093	BT2FRA093
Italy	2	AY823222	BT2ITA222
South Africa	3	AF512906	BT3ZAF906
Greece	4	AY691692	BT4GRC692
Israel	4	AY775158	BT4ISR158
Italy	4	AY775156	BT4ITA156
South Africa	8	AY120938	BT8ZAF938
Greece	9	AY449651	BT9GRC651
Italy	9	AY775161	BT9ITA161
South Africa	11	AF512921	BT11ZAF921
South Africa	18	AF512915	BT18ZAF915
Asia and Australia			
Indonesia	1	AF529049	BT1IDN049
Australia	2	AF529057	BT1AUS057
China	3	AF135224	BT2CHN224
Indonesia	4	AF529050	BT3IDN050
China	4	AF135225	BT3CHN225
China	5	AF135226	BT4CHN226
China	12	AF135227	BT12CHN227
China	16	AF135229	BT16CHN229
Australia	20	AF529055	BT20AUS055
Australia	21	AF529058	BT21AUS058
Indonesia	23	AF529051	BT23IDN051
India	12	KC662621	BT12IND621
India	9	KP696660	BT9IND660
India	23	EU131022	BT23IND022
India	1	KP696581	BT1IND581
India	16	KX302643	BT16IND643
India	4	KF560426	BT4IND426
India	3	JQ771822	BT3IND822
India	18	EU131025	BT18IND025
India	2	KX650180	BT2IND180
Epizootic hemorrhagic virus	1	NC_013405	EHD1USA405

The 46 BTV serotypes, including the epizootic hemorrhagic disease virus serotype 1 analyzed in the study were listed in the table. The following convention was used to identify nucleotide sequence-BT_serotype number_three-letter country code last three digits of sequence accession number. BTV=Bluetongue virus

## Results and Discussion

Sequence analysis of the NS3 gene revealed a coding region of 690 bp with two in-frame AUG codons. Upon alignment of nucleotide sequences, the region showed nucleotide sequence identities of 81-84% with North American (USA), 82-85% with Central American and Caribbean (Honduras, Costa Rica, Dominican Republic, Puerto Rico and Jamaica), and 83% with African and Mediterranean isolates (South Africa, Greece, Israel, France, and Italy). Despite their geographically distant origins, a high sequence homology of 90-96% was observed with Asian and Australian isolates. The nucleotide similarity shared with Asian and Australian strains support the hypothesis of a common origin of infection while the sequence variability observed with other BTV isolates demonstrated viral evolution over a period. As expected, a high rate of sequence identity (95-98%) was observed with BTV serotypes from the Indian sub-continent. The nucleotide substitutions may reflect the intrinsic rate of viral mutation (lack of proofreading activity of viral RNA-dependent RNA polymerase) or are the result of viral selection influenced by different vectors and hosts as previously demonstrated [15].

**Figure-1:**
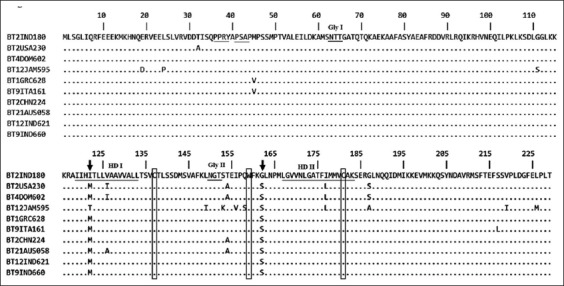
Alignment of the amino acid sequences of bluetongue virus (BTV) non-structural protein-3 (NS3) protein. Alignment of the deduced amino acid sequences (229 aa) of BTV NS3 protein. The late motif domains PPXY/PSAP, glycosylation sites (Gly I and Gly II) and hydrophobic domains (HD I and HD II) have been underlined. The unique amino acid substitutions isoleucine (I) and glycine (G) were indicated by an arrow. The conserved residues cysteine (C) and tryptophan (W) are indicated within a box.

Despite the host-geographic connection of sequence variability, amino acid sequence (229 aa) analysis demonstrated the stability of NS3 protein in the BTV2 strain circulating in India. The region showed high sequence homology of 93-99% at amino acid level with various BTV serotypes. We found that PPXY/PTAP late domain motifs, glycosylation sites (63-65aa and 150-152aa), the two hydrophobic domains (119-133aa and 167-183aa), the cysteine (C) residues at amino acid positions 137 and 181, and a tryptophan residue (W) at position 159 were conserved in NS3 protein (Figure-1). The nucleotide substitutions in the NS3 gene coding region were generally silent or conservative in deduced amino acid sequences, thus not affecting the protein structure. The likely explanation is in the key role of the protein in viral maturation and egress [7]. Furthermore, we found unique amino acid substitutions of isoleucine for methionine at position 122 and glycine for serine at position 162 in this highly conserved protein. The NS3 protein is non-essential for viral replication [16]; thus, nonsynonymous mutation in its gene could be less restrictive. However, the protein seems to be involved in the inhibition of interferon synthesis as well as in the ability of the virus to overcome the host immune response [17]. It would be interesting to understand the effect of these changes on the infectivity of BTV.

**Figure-2:**
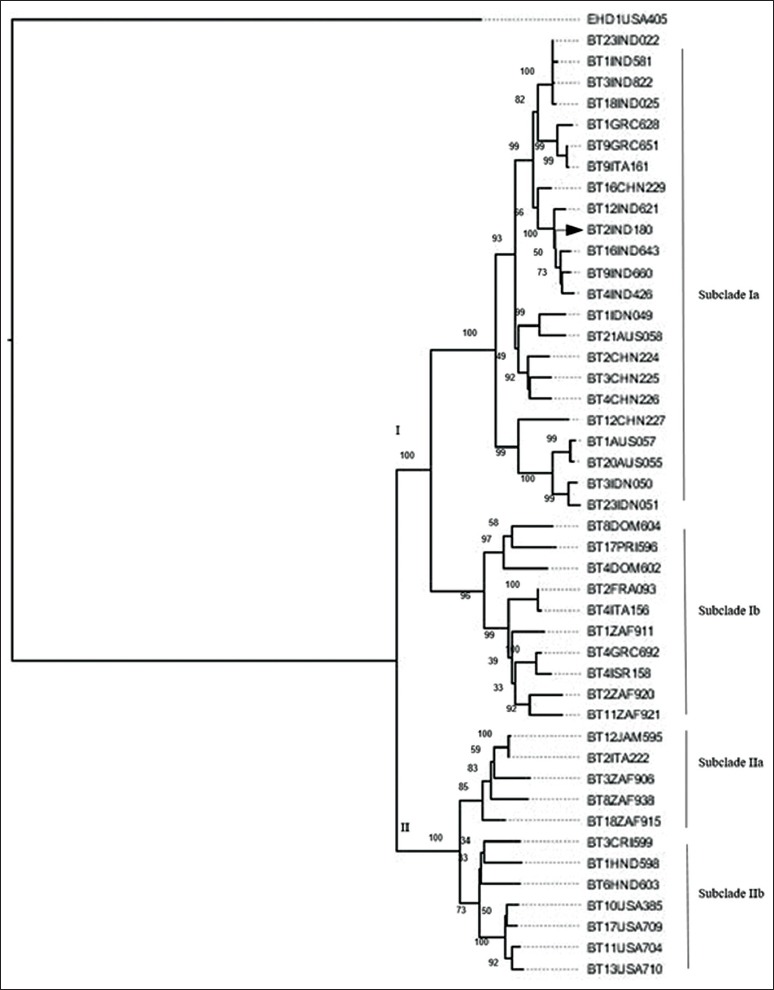
Phylogenetic tree of the non-structural protein-3 nucleotide coding sequences of different bluetongue virus (BTV) isolates of the world. The evolutionary history was inferred using the neighbor-joining method using MEGA7 with bootstrap values of 500 replicates. Different lineages and sub-lineages are indicated. Our isolate, Indian BTV serotype-2 is indicated by a triangle.

Computation of evolutionary distances among global BTV serotypes by maximum likelihood method resulted in recovery of two major Clades, I and II (Figure-2). Within these broad clades, four subclades (Ia, Ib, IIa, and IIb) or topotypes have been identified that were associated with respective geographical regions. These observations on the geographical distribution of BTVs are in agreement with previously defined topotypes [18]. The existence of distinct topotypes within individual BTV serotypes indicate significant geographically independent evolution of this segment. Geographically diverse group of isolates representing Central America (Dominican Republic and Puerto Rico), Mediterranean Basin (Greece, Italy, Israel and France) and South Africa were included in subclade Ib. Subclade IIa includes isolates from Mediterranean Basin (Italy), Caribbean (Jamaica), and South Africa. BTV isolates from the USA segregated in subclade IIb along with viruses from Central America (Costa Rica and Honduras). Recent BTV isolates from Greece (BT1GRC628 and BT9GRC651) and Italy (BT9ITA161) shared a closer relationship with Asian strains. Our observations support the earlier reports of Asian origin of recent European isolates as a result of recent climatic changes and the activity of *Culicoides* vector [19]. The BTV2 virus (BT2IND180) from India closely clustered with isolates of Asia (India, China, and Indonesia) and Australia in Subclade Ia. Phylogenetic clustering within subclade Ia suggested the exotic status of this serotype in India and its origin from Asia and Australia.

## Conclusion

Genetic characterization of circulating strains remains essential to understand whether the virus is evolving and to draw up surveillance and prevention strategies. In this study, NS3 gene of Indian BTV2 isolate was sequenced and analyzed in comparison with BTV sequences worldwide. Phylogenetic network analysis of 46 BTV sequences revealed four topotypes irrespective of their serotype. The NS3 gene sequence-based clustering indicated that the Indian BTV2 isolate is closely related to strains from Asia and Australia, representing a single virus lineage. Although the pattern of evolution of BTV2 isolate is different from other Indian BTV serotypes and global isolates, NS3 gene sequence and functional motifs are highly conserved.

## Authors’ Contributions

SD was the project leader. RSP and SD were responsible for experimental and project design. RSP performed most of the experiments. SR was involved in sequence analysis. MP made conceptual contributions. All authors participated in writing the manuscript and approved it for submission.
